# Oligomerization of a G protein-coupled receptor in neurons controlled by its structural dynamics

**DOI:** 10.1038/s41598-018-28682-6

**Published:** 2018-07-10

**Authors:** Thor C. Møller, Jerome Hottin, Caroline Clerté, Jurriaan M. Zwier, Thierry Durroux, Philippe Rondard, Laurent Prézeau, Catherine A. Royer, Jean-Philippe Pin, Emmanuel Margeat, Julie Kniazeff

**Affiliations:** 1 IGF, Univ Montpellier, CNRS, INSERM, Montpellier, France; 2 CBS, Univ Montpellier, INSERM, CNRS, Montpellier, France; 3 Cisbio Bioassays, F-30200 Codolet, France; 4 Department of Biological Sciences, Rensselaer Polytechnic Institute, Troy, NY 12180 USA

## Abstract

G protein coupled receptors (GPCRs) play essential roles in intercellular communication. Although reported two decades ago, the assembly of GPCRs into dimer and larger oligomers in their native environment is still a matter of intense debate. Here, using number and brightness analysis of fluorescently labeled receptors in cultured hippocampal neurons, we confirm that the metabotropic glutamate receptor type 2 (mGlu_2_) is a homodimer at expression levels in the physiological range, while heterodimeric GABA_B_ receptors form larger complexes. Surprisingly, we observed the formation of larger mGlu_2_ oligomers upon both activation and inhibition of the receptor. Stabilizing the receptor in its inactive conformation using biochemical constraints also led to the observation of oligomers. Following our recent observation that mGlu receptors are in constant and rapid equilibrium between several states under basal conditions, we propose that this structural heterogeneity limits receptor oligomerization. Such assemblies are expected to stabilize either the active or the inactive state of the receptor.

## Introduction

G protein-coupled receptors (GPCRs) are key players in neuronal communication, modulating synaptic transmission both at the pre- and post-synaptic levels. As such, they remain promising targets for the treatment of a number of brain diseases. These proteins, harboring 7 transmembrane helices, explore dynamically a complex conformational landscape^1^. Binding of different ligands (agonists, allosteric ligands, antagonists, etc.) shifts this conformational equilibrium in unique ways, enabling a defined subset of intracellular proteins to interact with the receptor^2^. Originally thought to function as monomers, GPCRs are now generally accepted to associate into homo- or heterodimers *in vitro* and in heterologous cells^3,4^, with a few cases validated in native tissues^5,6^. Such complexes of a wide range of GPCRs have been shown to display distinct properties in different cell types^3,4,7–11^, possibly a consequence of a modification of the conformational landscape due to protein-protein interaction. Furthermore, some studies even report the existence of larger complexes of more than two receptors^12–14^, but the reality of this phenomenon in native cells remains elusive. This is best illustrated for the GABA_B_ receptor that was reported to form tetramers and even larger complexes in heterologous cells and possibly in neurons, resulting in a modulation of the signaling efficacy^12,15,16^. To move forward, many fundamental properties of GPCR oligomers, including their stoichiometry, conformational heterogeneity, dynamics, localization and mechanisms of interaction must be defined essentially in native tissues.

Metabotropic glutamate (mGlu) receptors are receptors for the major excitatory neurotransmitter in the central nervous system (CNS) glutamate. They modulate synaptic signaling in the brain and constitute promising drug targets for several CNS disorders, including schizophrenia, depression and drug addiction^17,18^. mGlu receptors belong to the class C GPCRs, which are characterized by a large extracellular domain containing the orthosteric binding site. Furthermore they are constitutive dimers, linked by an extracellular disulfide bridge and this dimerization is necessary for agonist-mediated G protein activation^19^. In their basal state mGlu receptors form strict dimers in HEK293 cells and Xenopus oocytes^20,21^. However, it was recently shown that when the active state was stabilized it was possible to cross-link mGlu_2_ receptor dimers in HEK293 cells through their transmembrane (7TM) domain, something that was not possible in the basal state^22^. A plausible explanation for this observation is the ligand-dependent modulation of mGlu_2_ conformational dynamics. *In vitro* single molecule studies of the structural dynamics of both the isolated Venus flytrap (VFT) domain and the full length mGlu_2_ receptor have shown that the mGlu_2_ receptor is highly dynamic in presence of a non-saturating concentration of agonist, a state that mimics the basal state^23,24^. In contrast, saturating concentrations of agonist or antagonist stabilized specific conformations with reduced conformational dynamics. Together these observations suggest a possible link between reduced conformational dynamics and GPCR oligomerization.

In this study we aimed to determine the stoichiometry of the mGlu_2_ receptor in living neurons using a particle counting technique, 2-photon fluctuation microscopy with scanning number and brightness (sN&B) analysis. We compared the stoichiometry of mGlu_2_ in the basal state with that observed in presence of ligands or other conditions - mutations and cross-linking - which modulate the conformational equilibria between the active or inactive states. We found that in the basal state and in the physiological expression range, mGlu_2_ is mainly dimeric. However, when the conformational dynamics were reduced - either pharmacologically or through mutations - the receptor formed higher order oligomers. Our observations suggest a new concept for GPCR oligomerization, establishing a potential reciprocal link between the receptor conformational dynamics and its oligomeric state both of which can be modulated by ligand binding. Such a mechanism suggests that oligomerization stabilizes specific conformations of the receptor, facilitating either activation or inhibition.

## Results

### Overview of scanning number and brightness

To characterize the oligomeric state of the mGlu_2_ receptor in live neurons we used a fluorescence fluctuation microscopy method called 2-photon sN&B. First proposed by Gratton and co-workers^25^, sN&B is one of a family of techniques based on fluorescence fluctuation analysis. In these approaches, fluorescent particles diffuse in and out of a small effective observation volume, *V*_*eff*_, created inside the sample, yielding fluctuations in fluorescence intensity about the mean. Both the mean fluorescence, $\langle F\rangle$, and the variance, ${\sigma }^{2}$ (fluctuations) are calculated from the time trace of fluorescence intensity.

In general, for two samples displaying identical average fluorescence intensity, a sample containing a few bright molecules will exhibit significantly larger fluctuations than a sample containing a large number of dim ones. Consequently, a GFP-fusion protein that dimerizes will yield twice the molecular brightness (*ε*) of the corresponding monomers, as well as two-fold fewer diffusing particles (*n*) since they move as pairs. Then, the measured molecular brightness in the sample, normalized to that of the dye *ε*_*GFP*_, is a direct measure of the protein stoichiometry. Simultaneously, the fluorescence intensity reports on the GFP-protein receptor expression level.

In scanning Number and Brightness (sN&B), multiple (e.g. 100) rapid raster scans of imaging fields of view (FOV) provide multiple fluorescence intensity measurements at each pixel of the FOV, from which the <*F*> and *σ*^2^ at each pixel can be calculated, yielding spatial maps of number, *n*, and brightness, *ε*. While single pixel-based sN&B data can be rather noisy, and include both positive and negative brightness values, averaging over all pixels within an individual region of interest (the cell membrane, for example) provides robust values for the average true brightness (See also Supplementary Method).

### Basal state mGlu_2_ is a dimer in cultured hippocampal neurons

We applied sN&B to mGlu_2_ receptor expressed in live hippocampal neurons to determine its stoichiometry. Compared to resonance energy transfer (RET) -based methods (such as Förster RET (FRET) and lanthanide RET (LRET)), sN&B offers certain advantages. Notably, detection of association is independent of the distance between the fluorophores or their relative orientation. Thus, complexes that would be undetected by RET can be quantified by sN&B. In contrast, any structural information based on the proximity between fluorophores observed in RET is lost in an sN&B experiment as it only provides information about the association of various partners in a single diffusing complex. Finally, unlike single molecule methods, sN&B is compatible with a wide range of expression levels.

**Figure 1:**
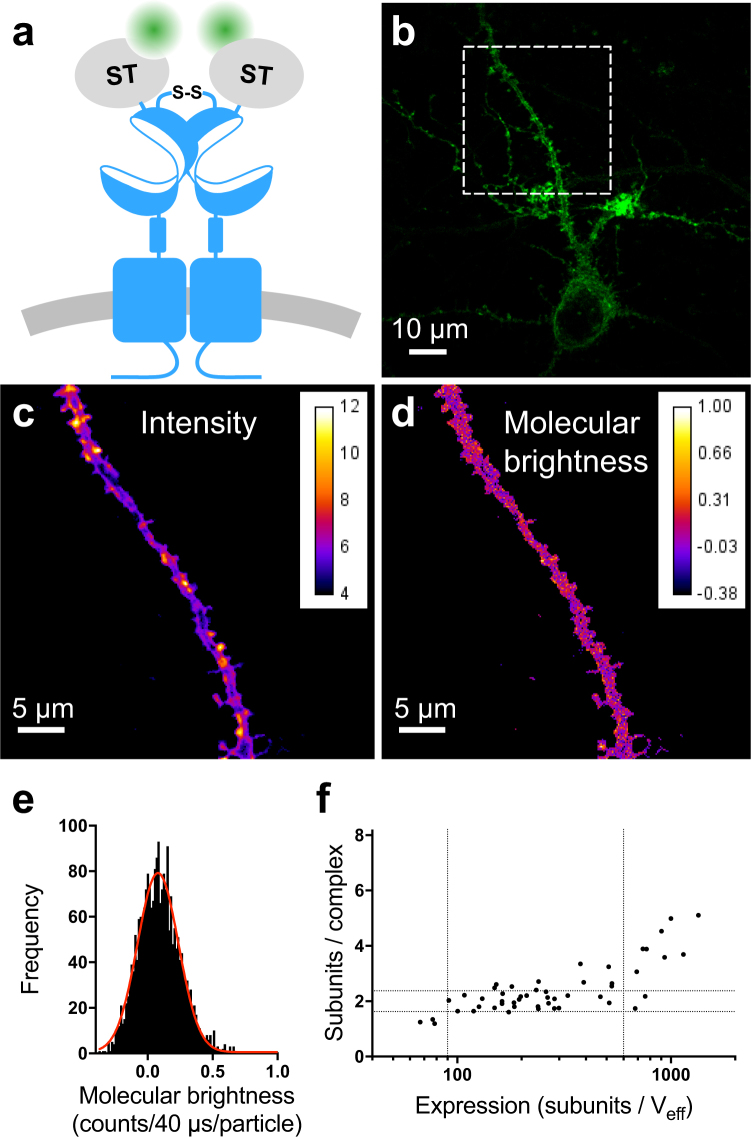
The mGlu_2_ receptor is mainly dimeric in cultured hippocampal neurons. **(a)** Schematic representation of the homodimeric mGlu_2_ receptor N-terminally SNAP-tagged (ST-mGlu_2_) and labeled with Alexa Fluor 488 (Alexa488). **(b)** Intensity image by two-photon microscopy of a primary neuron transfected with ST-mGlu_2_ labeled with Alexa488. The white dashed square represents the area selected for further imaging and analysis. **(c)** Average intensity map obtained from a stack of 100 images of the dashed square in **(b)** after thresholding. **(d)** Molecular brightness (*ε*, counts/40 μs/molecule) map of the dashed square in **(b)**. **(e)** Histogram of molecular brightness values corresponding to the map in **(d)**. The center of the distribution is estimated with a Gaussian fit (red line) giving *ε* = 0.0798 ± 0.0030 or 1.66 ± 0.06 subunits/complex (expression 98 subunits per effective volume (V_eff_ = 0.355 fL)). **(f)** Number of mGlu_2_ receptor subunits per complex as a function of subunits per V_eff_. The vertical dotted lines indicate three expression regimes with different complex sizes. The horizontal dotted lines indicate the molecular brightness of a dimer ± SD. Data are from 14 independent experiments.

For sN&B analysis we transfected cultured rat hippocampal neurons with plasmids encoding the mGlu_2_ receptor with a SNAP-tag fused to the extracellular N-terminus (ST-mGlu_2_, Fig. 1a). Our previous studies indicated that the SNAP-tag did not affect, in any way, the function of this receptor^20,26^. The SNAP-tag was covalently labeled with the cell-impermeable fluorophore Alexa488 immediately prior to imaging, providing a stable signal over time and allowing specific labeling of the entire cell-surface population of ST-mGlu_2_^12^. Fluorescence intensity images of dendrites or axons from living neurons expressing ST-mGlu_2_ showed a spatially heterogeneous distribution of the receptors (Fig. 1b,c). However, the molecular brightness map revealed that the receptor stoichiometry was rather homogeneously distributed in the field of view (Fig. 1d). For further quantification of the mGlu_2_ receptor stoichiometry we constructed histograms of the molecular brightness maps for each individual cell (Fig. 1e). The peak value of each histogram corresponds to the stoichiometry of the major population of receptors in a given neuron. Using the molecular brightness of free Alexa488 calibrated in viscous glycerol solution (0.048 ± 0.001 counts/40 μs/molecule, mean ± SEM, 48 measurements), the measured molecular brightness values of the Alexa488 labeled ST-mGlu_2_ complexes in neurons were converted to the number of mGlu_2_ subunits per complex under the assumption that all subunits were labeled with Alexa488. In addition, since the SNAP -tag on the mGlu_2_ receptors is extracellular, we do not expect the brightness of the dye to be influenced by the intracellular environment, especially since Alexa488 is rather insensitive to environmental differences. Receptor stoichiometry was determined for neurons spanning a wide range of expression levels, which we quantify by the number of subunits in the V_eff_ (Fig. 1f). The neurons exhibited three expression regimes. In the expression range between 90 and 600 subunits/V_eff_ the ST-mGlu_2_ stoichiometry was rather constant and equaled on average 2.2 ± 0.4 subunits per complex (mean ± SD, 37 cells). This indicates that the major population of mGlu_2_ receptors in this expression range is dimeric, an observation in agreement with previously published findings in HEK293 cells and Xenopus oocytes^20,21^. Above 600 subunits/V_eff_, the stoichiometry of the complexes increased with increasing expression. The formation of these higher order oligomers at higher expression levels has not been previously reported. Finally, in the range below 90 subunits/V_eff_, the stoichiometry appeared to be lower than dimer, possibly due to the formation of dimers between ST-mGlu_2_ and endogenously expressed receptors that are not labeled. As mentioned above, the intensity map of individual cells was heterogeneous. The 10% highest intensity pixels formed spatially segregated clusters (Supplementary Fig. S1a–c). The molecular brightness calculated for these pixels was comparable to that observed for pixels outside clusters with similar expression levels (Supplementary Fig. S1d). Thus, while there appeared to be some clustering of the receptor, this did not result in higher oligomerization probability.

**Figure 2:**
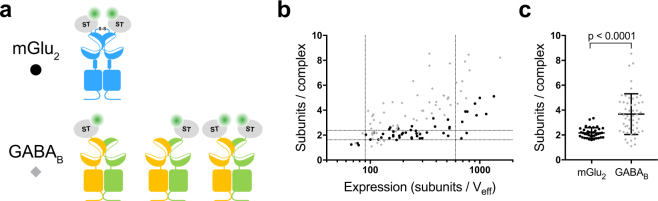
The GABA_B_ receptor forms high order oligomers in cultured hippocampal neurons. **(a)** Schematic representation of the mGlu_2_ receptor and the three combinations of untagged and SNAP-tagged GABA_B_ receptor subunits. **(b-c)** Comparison of the subunits per complex for all GABA_B_ receptor combinations (grey) with the mGlu_2_ receptor (black) as a function of the expression level **(b)** or for cells with an expression in the 90–600 subunits/V_eff_ range **(c)**. The number of subunits per complex is higher for the GABA_B_ receptor than for the mGlu_2_ receptor (p < 0.0001; unpaired, two-sided t-test). Error bars represent SD. Data are from 14–17 independent experiments.

To underscore the accuracy of sN&B for quantifying the stoichiometry of cell surface receptors in primary neurons, we performed similar experiments on another class C GPCR, the GABA_B_ receptor, which is composed of two subunits, GABA_B1_ and GABA_B2_, both required for proper targeting of a functional receptor to the cell surface^27,28^. The GABA_B_ receptor is known to form oligomers in heterologous expression systems and most likely in native tissue, as well^12,15,16^. We took advantage of the fact that the GABA_B_ receptor is an obligatory heterodimer by labeling either only one of the subunits or both (Supplementary Fig. S2a). While the observed number of fluorophores was higher for the construct where both subunits were labeled (Supplementary Fig. S2b, blue), the molecular stoichiometry distributions of the three differently tagged GABA_B_ receptors, corrected for the theoretical number of fluorophores per heterodimer, were the same within experimental uncertainty (Supplementary Fig. S2c). This consistency reflects both the reliability of the sN&B method for stoichiometry measurement, and the fact that the SNAP-tag labeling efficiency is close to 100%. Interestingly, in contrast to the mGlu_2_ receptor, the GABA_B_ receptor displayed an average stoichiometry ranging from 2 to 8 per diffusing complex depending on the expression level (Fig. 2). Hence, in living neurons, sN&B analysis yields stoichiometries of the GABA_B_ and mGlu_2_ receptors that are consistent with previous findings on heterologous expression systems^12,15,16^.

### mGlu_2_ expression in transfected neurons is comparable to that of native receptors

**Figure 3:**
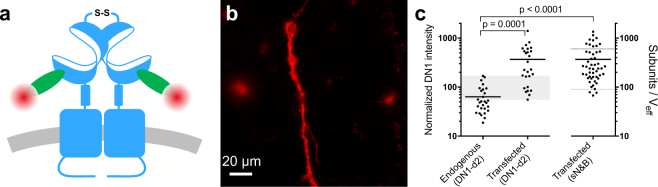
Overlap between expression levels of mGlu_2_ in native and transfected neurons. Expression of mGlu_2_ receptor in native and transfected primary hippocampal neurons was measured by immunofluorescence using a d2-labeled single domain antibody (DN1-d2) specific for the mGlu_2_ receptor. **(a)** Schematic drawing of DN1-d2 labeling of the mGlu_2_ receptor. **(b)** Image of a DN1-d2 labeled native neuron with endogenous expression of mGlu_2_ receptor by epifluorescence microscopy. **(c)** Mean intensity of DN1-d2 labeling of neurons for endogenous (28 cells) or transfected (26 cells) mGlu_2_ receptor expression. To compare the expression of endogenous mGlu_2_ receptors with the expression levels obtained in N&B experiments, the DN1-d2 mean intensity in transfected neurons was normalized to the mean number of mGlu_2_ subunits per effective volume (V_eff_) in transfected neurons determined with sN&B. The standard deviations of the transfected receptor densities in the DN1-d2 labeling and the sN&B experiments were similar after normalization (320 and 310 subunits/V_eff_, respectively). The endogenous mGlu_2_ expression overlaps with the expression in transfected neurons in the 55–170 subunits/V_eff_ range (shaded area), whereas the mean expression is higher in transfected neurons (p = 0.0001 (DN1-d2) or p < 0.0001 (sN&B), one-way ANOVA, Bonferroni’s multiple comparisons test). Data are from 4 (DN1-d2) or 14 (sN&B) independent experiments.

Because the oligomerization of the mGlu_2_ receptor increased with increasing expression in transfected neurons, it was important to compare the receptor expression levels after transfection with those of endogenous mGlu_2_ receptors. Accordingly, we used a single domain antibody (sdAb) specific to the mGlu_2_ receptor (DN1)^29^ labeled with the far red dye d2 that allowed us to detect both endogenous and transfected receptors (Fig. 3a). DN1 is specific of mGlu_2_ among the 8 mGlu receptors^29^ including the closely related mGlu_3_ (65% of identity and 83% of similarity). We measured the fluorescence intensity from DN1-d2 labeling of native hippocampal neurons using epifluorescence microscopy (Fig. 3b), and compared the distribution of average DN1-d2 intensities for each neuron to that of neurons transfected with ST-mGlu_2_ (Fig. 3c). Both the native and transfected neurons exhibited rather broad distributions of expression levels with significant overlap between the expression levels in native and transfected neurons. The mean mGlu_2_ receptor expression was found to be only 5.8 ± 1.2 times higher in transfected neurons than in neurons natively expressing the mGlu_2_ receptor. This indicates that transfection does not lead to large over-expression and that interference from endogenous mGlu_2_ receptors in the sN&B analysis of mGlu_2_ receptor stoichiometry is expected to be minimal, with the exception of the lowest expression levels. Since the neuronal transfections were performed under the same conditions for DN1-d2 labeling and sN&B experiments, we assumed that the transfected neurons in sdAb labeling experiments and in sN&B experiments expressed the same average levels of mGlu_2_ receptor. This was further supported by the two intensity distributions having similar coefficients of variation (86% and 83%) (Fig. 3c). Thus, we normalized the average intensity measured on DN1-d2 labeled transfected neurons to the average intensity value determined by sN&B (Fig. 3c). Using this calculation, endogenous mGlu_2_ expression in hippocampal neurons was found to range from 20 to 170 subunits/V_eff_. This range partly overlaps with the expression levels in which we found the major mGlu_2_ population to be dimeric (90 to 600 subunits/V_eff_). Altogether these results suggest that endogenous mGlu_2_ receptors in hippocampal neurons exist predominantly as dimers in their basal state.

### Ligand binding favors receptor oligomerization

**Figure 4:**
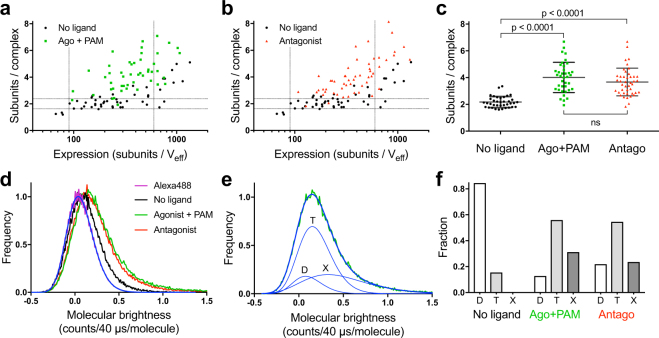
Ligands favor mGlu_2_ oligomerization. **(a**,**b)** Comparison of the mGlu_2_ receptor stoichiometry as a function of its expression level in the absence of ligand (black) or when bound to agonist and PAM (10 nM LY379268 + 3 μM BINA, green, a) or to antagonist (400 nM LY341495, red, b). **(c)** Between 90 and 600 subunits/V_eff_, the mean mGlu_2_ stoichiometry is 2.2 ± 0.4, 4.0 ± 1.1 and 3.7 ± 1.0 subunits/complex in the absence of ligand, when bound to agonist and PAM or antagonist, respectively (mean ± SD). One-way ANOVA followed by Bonferroni’s multiple comparisons test was used to show that the increased stoichiometries were statistically significant. **(d)** Normalized accumulation of molecular brightness histograms for monomeric Alexa488 (purple) overlaid with a log-normal fit (blue) and for all neurons with expression in the 90–600 subunits/V_eff_ range in absence of ligand (black) or bound to agonist and PAM (green) or antagonist (red). **(e)** Example of a fit of the accumulated histogram for mGlu_2_ bound to agonist and PAM in neurons with expression in the 90–600 subunits/V_eff_ range (from panel d, green). The total fit (thick blue curve) is the sum of three log-normal fits (thin blue curves) corresponding to the population of dimers (D), tetramers (T), and oligomers larger than tetramers (X). The peak values of the dimer and tetramer fits are fixed to multiples of the peak value of monomeric Alexa488. The peak value for larger oligomers was unconstrained. **(f)** Fraction of the different oligomeric species (D, T and X) under the given conditions calculated from the areas under the individual fit curves. Data are from 11–14 independent experiments.

Although dimerization of mGlu receptors is stabilized mainly by hydrophobic interactions and an inter-subunit disulfide bridge between the VFTs, the association of receptor dimers into larger oligomers is thought to occur via seven-transmembrane (7TM) domain contacts^22^. Since ligand binding is known to modify the 7TM interface between the subunits in the dimer^22^, ligands could affect receptor oligomerization properties, as well. To investigate this possibility, we measured by sN&B, the stoichiometry of ST-mGlu_2_ in transfected hippocampal neurons in presence of an mGlu_2/3_ selective agonist LY379268^30^ in combination with the mGlu_2_ selective positive allosteric modulator (PAM) BINA^31^ (Fig. 4a). Because no agonist selective for mGlu_2_ versus mGlu_3_ is commercially available, we used 10 nM of the mGlu_2_/mGlu_3_ agonist LY379268 (close to its K_i_ value for mGlu_2_ measured in rat brain membrane against LY341495 (14 nM^32^)) and 3 μM of BINA (around 10 fold its K_i_ value for mGlu_2_ (305 nM^33^)) in order to limit mGlu_3_ activation and to reach full activation of mGlu_2_ thanks to the PAM. We found that even at expression levels at which the mGlu_2_ receptor remained dimeric in basal conditions (between 90 and 600 subunits/V_eff_), the activated receptor stoichiometry was significantly higher and increased with increasing expression. Surprisingly, a similar increase in receptor stoichiometry was observed in the presence of a saturating concentration of the non-selective competitive antagonist LY341495^30,34^ (Fig. 4b). Quantification of the major mGlu_2_ population revealed a similar shift to larger complex sizes when bound either to agonist and PAM or to antagonist (no ligand: 2.2 ± 0.4 subunits/complex (37 cells), agonist + PAM: 4.0 ± 1.1 subunits/complex (38 cells), antagonist: 3.7 ± 1.0 subunits/complex (43 cells)) (Fig. 4c). As in the absence of ligand, the highest intensity pixels were located in spatially segregated clusters (Supplementary Fig. S3).

The observed increase in receptor stoichiometry upon ligand binding was assessed by calculating the peak value of each brightness histogram, which represents the major oligomer population. However, additional minor populations are likely to be present. Thus, in order to better quantify the heterogeneity in oligomeric species present in the neurons under the different conditions, we constructed molecular brightness histograms from all cells in our dataset with an expression between 90 and 600 subunits/V_eff_. As expected, the accumulated histogram for the mGlu_2_ receptor in absence of ligand was shifted to higher values of molecular brightness compared to monomeric Alexa488 in solution, and even higher when mGlu_2_ was bound to either agonist and PAM or antagonist (Fig. 4d). We quantified the fraction of dimeric, tetrameric, and higher order species by fitting each histogram to a sum of log-normal distributions representing the different numbers of mGlu_2_ subunits per complex (Fig. 4e and Supplementary Fig. S4). Under basal conditions ∼80% of the mGlu_2_ receptors were dimeric, whereas when the receptors were bound to either agonist and PAM or antagonist we found ∼60% tetramers, ∼20–30% higher order species and only ∼10–20% dimers (Fig. 4f). While the mGlu_2_ receptor was mainly dimeric under basal conditions in the expression range between 90 and 600 subunits/V_eff_, at expression levels above 600 subunits/V_eff,_ the accumulated histograms showed a proportion of complexes larger than dimers (Supplementary Fig. S4). Indeed, in the expression range above 600 subunits/V_eff_ and under basal conditions only ∼20% of mGlu_2_ receptors were dimeric (Supplementary Fig. S4g,j). This trend was exacerbated in presence of either agonist and PAM or antagonist (Supplementary Fig. S4h–j).

### mGlu_2_ C-terminal domain does not influence oligomerization

Intracellular scaffolding proteins are known to link membrane proteins and to promote complex formation^35,36^. Because sN&B analysis detects co-diffusing species, we wondered whether scaffolding proteins promoted co-diffusion of mGlu_2_ receptor dimers or oligomers in the membrane without interaction between the receptors. To test this possibility, we carried out sN&B experiments on a truncated version of the receptor where the 26 C-terminal amino acids were deleted, mGlu_2_-Δ846, to limit the potential for scaffold protein binding. This deletion did not affect the function of the receptor (Supplementary Fig. S5a). The oligomerization distributions determined by sN&B for mGlu_2_-Δ846 both in absence of ligand and bound to agonist and PAM were found to be similar to those of the wild type receptor (Supplementary Fig. S5b–d), demonstrating that the formation of higher order oligomers of mGlu_2_ is independent of the distal C-terminal region of the receptor, and thus its interaction with scaffolding proteins.

### mGlu_2_ oligomerization interfaces are different in the active and inactive states

**Figure 5:**
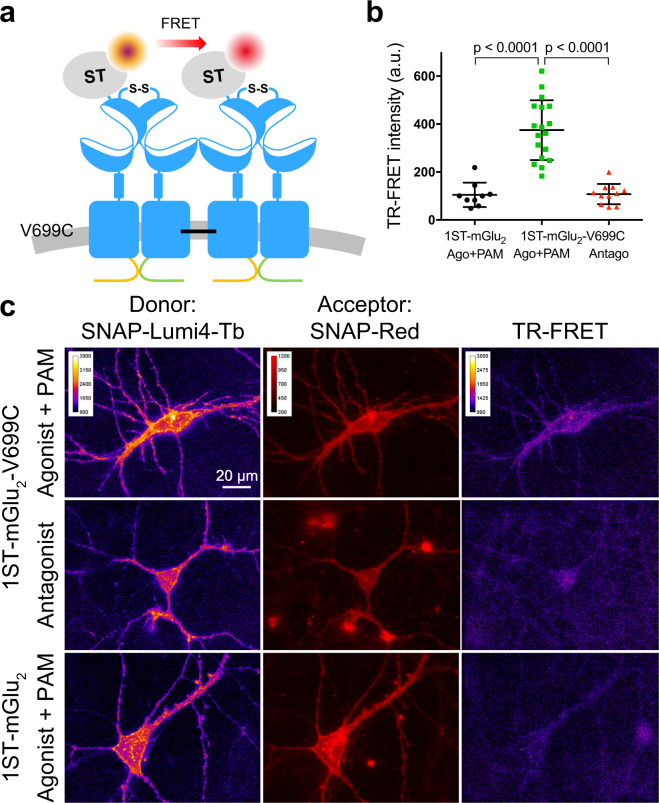
mGlu_2_ dimers are in close physical proximity in the active state. LRET between mGlu_2_ dimers with a cysteine introduced in TM4 (V699C) that can form disulfide bonds between dimers upon incubation with the oxidizing agent copper phenanthroline (CuP) when the receptor is in the active state. **(a)** Schematic drawing of the experimental set up. A quality control system based on modified GABA_B_ C-terminal domains was used to ensure that each mGlu_2_ dimer only contained one SNAP-tag (1ST-mGlu_2_). LRET was measured between SNAP-Lumi4-Tb (donor) and SNAP-Red (acceptor) covalently attached to the SNAP-tags. **(b)** Scatter plot of the TR-FRET intensity in the low expression range (dashed lines in Supplementary Fig. S6). In this interval the TR-FRET intensity for 1ST-mGlu_2_-V699C incubated with CuP in presence of agonist and PAM (10 nM LY379268 and 3 μM BINA) is significantly higher than when the same receptor mutant is incubated with CuP in presence of antagonist (400 nM LY341495) or the negative control 1ST-mGlu_2_ incubated with CuP in presence of agonist and PAM (p < 0.0001, one-way ANOVA, Bonferroni’s multiple comparisons test). Data are from 4 (1ST-mGlu_2_-V699C+ agonist and PAM) or 2 (controls) independent experiments. **(c)** Representative donor, acceptor and TR-FRET images of 1ST-mGlu_2_-V699C incubated with CuP in presence of either agonist and PAM or antagonist, and 1ST-mGlu_2_ incubated with CuP in presence of agonist and PAM.

To further establish that the ligand-induced mGlu_2_ oligomerization observed with sN&B analysis is due to a direct and physical interaction between mGlu_2_ dimers we used disulfide cross-linking in combination with LRET. We have previously shown cross-linking of mGlu_2_ dimers in HEK293 cells in presence of a saturating concentration of agonist and PAM upon introduction of a cysteine in TM4 at residue 699 (V699C)^22^. Interestingly, in this prior study, the dimers could not be cross-linked in presence of a saturating concentration of antagonist, indicating that the oligomerization interfaces are different for the active and the inactive conformations. To confirm these observations in living neurons, we measured LRET between mGlu_2_ dimers using time-resolved FRET (TR-FRET) microscopy^37^ in neurons transfected with plasmids encoding mGlu_2_-V699C and incubated with the oxidizing agent copper phenanthroline (CuP) to facilitate cross-linking of dimers (Fig. 5a). In addition, a single subunit per dimer was SNAP-tagged such that labeling with a mixture of the LRET pair SNAP-Lumi4-Tb and SNAP-Red allowed observation of TR-FRET exclusively between dimers. The presence of only one SNAP-tag per dimer was ensured by replacing the C-terminal domains of SNAP-tagged and non-SNAP-tagged receptors with a quality control system based on modified C-terminal domains from the two GABA_B_ receptor subunits (called C1_KKXX_ and C2_KKXX_)^38^. Using this system, homodimers are retained in the ER and thus only mGlu_2_-V699C-C1_KKXX_/ST-mGlu_2_-V699C-C2_KKXX_ heterodimers (1ST-mGlu_2_-V699C) can reach the cell surface.

We observed a significantly higher TR-FRET signal for 1ST-mGlu_2_-V699C when cross-linking was carried out in the presence of agonist and PAM than in the presence of antagonist (Fig. 5b,c, Supplementary Fig. S6). The TR-FRET signal for non-mutated 1ST-mGlu_2_ was similar to 1ST-mGlu_2_-V699C in presence of antagonist, indicating that dimer-dimer cross-linking through TM4 was specific to the active conformation of the mGlu_2_ receptor. In sN&B experiments cross-linking ST-mGlu_2_-V699C did not change the distribution of complex sizes sufficiently to differentiate it from the wild type (data not shown), likely because of a low efficiency of cross-linking, as previously observed in HEK293 cells^22^ and which was probably exacerbated here by a reduction in concentration and incubation time with CuP to avoid toxicity to the neurons. As a result, the small population of cross-linked receptors would have only a minor contribution to the signal in sN&B experiments that monitors simultaneously all receptors. In contrast, in LRET experiments, the TR-FRET signal arises only from receptors that are close enough to transfer energy while the others do not contribute to the specific signal. Accordingly the small population of cross-linked receptors is sufficient to be detected by LRET as illustrated in Fig. 5b,c. While both antagonist and agonist and PAM treatments promoted oligomerization as revealed by sN&B experiments, only agonist and PAM application during the cross-linking step elicited TR-FRET signal using 1ST-mGlu_2_-V699C mutant. These LRET results confirm those observed in HEK293 cells, namely that the oligomerization interfaces for the active and the inactive conformations are different. In addition, the ability to cross-link mGlu_2_ dimers when stabilized in the active state indicates that the dimers are directly interacting within the higher order oligomers.

### Reducing conformational dynamics promotes oligomerization

**Figure 6:**
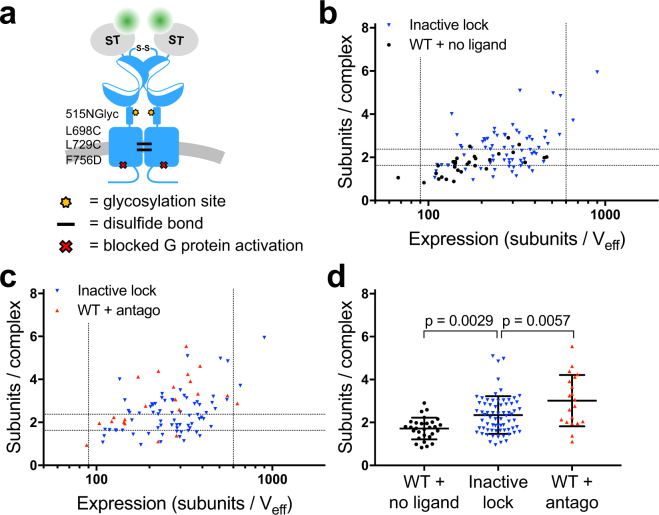
Locking the inactive conformation of mGlu_2_ allows its oligomerization in absence of ligand. (**a**) Schematic drawing of the mutations introduced to lock the inactive conformation of the mGlu_2_ receptor. An N-glycosylation site was introduced at residue 515 (515NGlyc) in the CRD to inhibit contact between the CRDs of the dimer and cysteines were introduced in TM4 and TM5 (L698C and L729C) to cross-link the inactive interface of the 7TM dimer. In addition a mutation in intracellular loop 3 that inhibits G protein activation (F756D) was introduced. (**b,c**) Stoichiometry as a function of the expression for mGlu_2_ locked in the inactive conformation compared with wild type receptor incubated with CuP and subsequently imaged in the absence of ligand (**b**) or bound to antagonist (**c**). (**d**) The number of subunits per complex for neurons with expression in the 90–600 subunits/V_eff_ range. In this range the mean receptor stoichiometry is 2.3 ± 0.9 subunits/complex for mGlu_2_ locked in the inactive conformation (70 cells), 1.7 ± 0.5 subunits/complex for wild type mGlu_2_ in the absence of ligand (29 cells), and 3.0 ± 1.2 subunits/complex for wild type mGlu_2_ bound to antagonist (20 cells). The mean stoichiometry of mGlu_2_ locked in the inactive conformation is significantly higher than wild type mGlu_2_ in the absence of ligand (p = 0.0029, one-way ANOVA, Bonferroni’s multiple comparisons test). Note that these apparent stoichiometry values are lower in the presence than in the absence of CuP (Supplementary Fig. S8). Errors and error bars are SD. Data are from 3–6 (mGlu_2_) or 10 (inactive lock) independent experiments.

The surprising finding that both activating and inactivating ligands promote an increase in mGlu_2_ oligomerization may be explained by the tendency of these ligands to reduce receptor conformational heterogeneity. Indeed, in the basal state of the receptor rapid transitions between active and inactive conformations have been observed that are dampened by binding of either agonists or antagonists^23,24^. It is thus possible that the stabilization of the receptor into one of the two ligand-specific dimeric states (agonist or antagonist) might be sufficient to promote higher-order oligomer formation, albeit via distinct interfaces. To explore this possibility we constructed a mutant receptor where both the extracellular domains (ECDs) and the 7TM domains were locked in the inactive conformation independently of ligand binding. To lock the ECDs, which includes both the VFTs and the cysteine rich regions, we introduced an NXS motif, which undergoes N-linked glycosylation in the ER, at position 515–517. This mutation has been shown to prevent the ECD dimer from reaching the active conformation^26^. To lock the 7TM domains, Leu698 in TM4 and Leu729 in TM5 were mutated to cysteines, thus allowing disulfide cross-linking of the inactive interface of the 7TM domains in the mGlu_2_ dimer upon addition of CuP^22^ (Fig. 6a). In addition, G protein activation was blocked by mutating Phe756 in the third intracellular loop to aspartate (F756D)^26^. We confirmed that the mutant receptor was locked in an inactive conformation using a TR-FRET sensor that monitors the conformational changes in the mGlu ECD during activation^26^ (Supplementary Fig. S7a,b). In addition, no functional response (G protein activation) was detected for the locked mGlu_2_, with or without the F756D mutation that prevents G protein activation (Supplementary Fig. S7c,d).

sN&B experiments with these mGlu_2_ receptors locked in the inactive conformation showed a stoichiometry that was significantly higher than the wild type receptor in absence of ligand (Fig. 6c,d). Although the oligomeric state of this mutant and crosslinked receptor remained lower than the wild type receptor bound to antagonist, this is likely due to the low efficiency of cross-linking under our experimental conditions. Of note, a decrease of ∼20% in the molecular brightness after incubation with CuP was observed for wild type receptors both with and without antagonist bound (Supplementary Fig. S8). Consequently, the locked receptors were only compared with sN&B data from wild type receptors incubated with CuP. It is unclear whether this decrease in brightness is due to an effect of CuP on the mGlu_2_ receptor stoichiometry or on the molecular brightness of the fluorophore. Taken together, these results reveal that when the conformational dynamics of the mGlu_2_ receptor are constrained in the absence of ligands, the receptor has a higher propensity for oligomerization, supporting the notion that oligomerization is favored by reduced conformational dynamics.

## Discussion

The present study shows that mGlu_2_ receptors are strict dimers under basal conditions when expressed in cultured hippocampal neurons at or near their endogenous levels as revealed using sN&B analysis of fluorescently labeled receptors. However larger complexes are observed when the receptor is activated with an agonist and a PAM, or inhibited with a competitive antagonist, as well as at higher expression levels. We show that these higher order complexes likely represent directly interacting mGlu_2_ dimers because: 1) limiting the interaction with scaffolding proteins by truncation of the distal part of the C-terminal tail of the receptor does not influence the complex formation; 2) a direct cross-linking of mGlu_2_ dimers via their 7TM domain can be observed, at least in the presence of agonist and PAM; and 3) mGlu_2_ dimers can form larger complexes in the absence of ligands when the receptor is locked in one conformation. This latter point also suggests that the basal conformational dynamics and thus the conformational heterogeneity of the receptor disfavors oligomerization in the basal state.

The ability of GPCRs to associate into dimers or larger oligomers was demonstrated more than 15 years ago^3^, but the physiological relevance of this phenomenon remains a topic of discussion^39,40^. Oligomerization has been reported to modulate ligand binding, signaling efficacy or cellular trafficking^3–5,10^. However, most of these observations were made in transfected cell lines under conditions of uncontrolled or strong over-expression, calling into question the functional significance of GPCR oligomerization at low expression levels in native environments. Recent studies examining GPCR oligomers at the single molecule level, when receptors are expressed at low density, consistently revealed transient receptor dimers, with increasing proportion of dimers and larger oligomers at higher receptor density^12,41,42^, again underscoring the question of the physiological significance of higher order GPCR oligomers.

In the present study, we applied sN&B, a fluorescence fluctuation-based technique particularly well-adapted to low concentrations of molecules, to analyze close to their physiological expression levels the oligomerization and stoichiometry of class C GPCRs in living neurons. The amplitude of the detected fluorescence fluctuations reports on the number of fluorescently labeled proteins within a diffusing complex, thus allowing the determination of its stoichiometry. This is in contrast to RET measurements traditionally used to analyze GPCR oligomerization which reveal proteins in close proximity with no indication of the stoichiometry. In addition, as compared to single molecule experiments, sN&B permits working at higher (“small ensemble”) expression levels. Finally, sN&B generates full pixel-by-pixel stoichiometry or interaction maps from the images, an advantage compared to Photon Counting Histogram (PCH) or Spatial Intensity Distribution Analysis (SpIDA) approaches.

Here, sN&B measurements on fluorescently labeled mGlu_2_ receptors in live neurons revealed that at expression levels comparable to the endogenous levels, the receptors are strict dimers, consistent with their constitutive and covalent association in homodimers^43^, and in agreement with previous studies in heterologous cells^20,21^. Furthermore, the heterodimeric GABA_B_ receptor was found to associate into larger complexes already at low expression levels and this higher order association was enhanced at higher surface densities, consistent with multiple studies both in transfected cells and in brain membranes^12,15,16^. The consistency between these previous observations and the present data suggests that the co-diffusing complexes observed with sN&B in living neurons correspond to real receptor dimers and oligomers with direct contact between the subunits and stable enough to allow their detection. To our knowledge this constitutes the first quantification of GPCR oligomerization in living neurons.

Interestingly, even at near endogenous expression levels, we observed the formation of higher order oligomers both when the mGlu_2_ receptor was activated with a combination of an agonist and PAM, and when the receptor was inhibited by a competitive antagonist. Modulation of GPCR oligomerization by agonists, antagonists and inverse agonists has been proposed over the last two decades^3,13,44,45^. However, the methods traditionally used to study GPCR oligomerization, such as co-immunoprecipitation, FRET and bioluminescence resonance energy transfer (BRET), are unable to distinguish between a structural rearrangement of the complex and an actual change in oligomerization^46^. Thus far, only a few studies of the effect of ligands on GPCR oligomerization were performed with methods capable of detecting a change in stoichiometry and all of them used transfected heterologous cell lines. For instance, in two independent studies using either FCS or SpIDA^13,44^, the oligomerization of the muscarinic M_1_ receptor was shown to be promoted by antagonist binding. The oligomerization state of the serotonin 5-HT_2C_ receptor was also shown to be regulated by antagonist binding using SpIDA, but unlike the M_1_ receptor, the 5-HT_2C_ receptor changed from being mainly dimeric to mainly monomeric upon antagonist binding^14^. Conversely the dimeric population of dopamine D_2_ receptor was shown by single molecule imaging to be unaffected by antagonists, but increased by agonist binding^47^. Based on FRET analysis^48^, the β2 adrenergic receptor was reported to form complexes up to tetramers upon inverse agonist binding when reconstituted in lipid vesicles, although the possibility of a rearrangement within the complexes rather than the formation of larger complexes could not be excluded. Of note, none of these observations were confirmed in a native environment.

**Figure 7:**
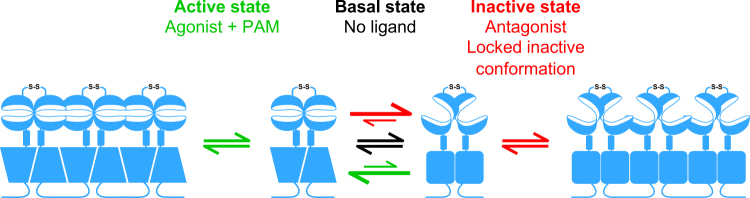
Structural stabilization promotes mGlu_2_ oligomerization. Model for mGlu_2_ receptor oligomerization. In the basal state (black arrows), there is a dynamic equilibrium between different conformational states. When a given conformational state of the receptor is stabilized, e.g. by binding of agonist and PAM (active state, green arrows) or antagonist (inactive state, red arrows) it increases the probability that the receptor can interact with other receptors in the same state, given the fact that the interdimeric interfaces are different in the active and the inactive states.

Although these prior studies point to a possible influence of receptor conformation on the formation of GPCR oligomers, we found, surprisingly that both inactivated and activated mGlu_2_ receptors associated into higher order oligomers in living neurons at endogenous expression levels. A possible explanation could be that the receptor adopts a specific conformation in the basal state that disfavors higher order oligomerization, and that is distinct from the active and inactive states. Alternatively, the recent observations that mGlu receptors are in a constant equilibrium between active and inactive states^23,24^ provide the basis for another explanation. The large movements observed between the VFTs in mGlu dimers at the sub-millisecond time scale are likely associated with the rotation of one of the 7TM domains in the dimer relative to the other. Indeed, based on cross-linking experiments performed in a heterologous expression system and on molecular modeling, the dimerization interface of mGlu receptor at the 7TM level was proposed to change according to the activation state of the receptor^22^. In the inactive state, the dimerization interface is composed of TM4 and TM5, leaving TM6 free to interact with the TM6 from another dimer, thus promoting oligomerization. Conversely, in the active state, dimerization occurs via TM6, thus the TM4–5 interface is available for association between dimers and could represent the oligomerization interfaces in the presence of agonist and PAM. This mechanism is further supported by the present results obtained in neurons demonstrating the ability to cross-link active dimers through cysteines introduced in TM4 only in the presence of agonist and PAM. In the basal state, mGlu receptor dimers are in a constant equilibrium between the active and inactive conformations, each involving different and mutually exclusive 7TM interfaces within the dimers. We propose that this conformational heterogeneity limits the oligomerization of the receptors in the basal state. Indeed, due to the constant oscillation between various conformational states, the proportion of receptor dimers able to associate is limited. Upon binding ligands, either agonist or antagonist, the conformational equilibrium is displaced towards one of the two extreme conformations, which favors self-oligomerization through distinct interfaces involved in the interaction between dimers. Thus, by decreasing the conformational heterogeneity, the ligands increase the relative proportion of receptor oligomers (Fig. 7).

Our hypothesis is supported by our experiments, in which decreasing the conformational heterogeneity using a set of mutations aimed at stabilizing the receptor in its inactive conformation led to an increase in mGlu_2_ oligomerization. Of interest, the VFT dimer of the GABA_B_ receptor has been proposed to undergo smaller conformational changes compared to mGlu receptors^49,50^. This potentially smaller conformational heterogeneity could explain why the GABA_B_ receptor can form larger oligomers without ligand at low receptor density^12,15,51^. Whether what we observed with the mGlu_2_ receptor can also occur with other GPCRs remains to be determined. Ligand-induced oligomerization would require that the oligomerization interface is masked or perturbed in a subset of the conformational states that a receptor samples in its basal state and may largely depend on the structural dynamics of the receptor. Recent studies already indicate that some GPCRs, such as the β2 adrenergic receptor^52–54^ also constantly oscillate between various states under basal conditions. These observations may provide a basis for understanding why, as described above, some ligands, either agonists or inverse agonists also facilitate the formation of oligomers^13,44,47,48^.

According to our proposed model for mGlu_2_ oligomerization, the cooperative formation of higher order oligomers would occur only between active dimers or between inactive dimers. As a consequence, the formation of these structurally distinct oligomers implies that either the active or the inactive conformation of mGlu dimers will be favored upon oligomerization. Accordingly, such mGlu_2_ oligomerization is expected to have consequences on the kinetics and duration of mGlu_2_-mediated signaling. Further work is needed to validate and quantify this hypothesis through the use of either mutant receptors unable to oligomerize, or other strategies allowing the control of this process.

In conclusion, our results confirmed that the mGlu_2_ receptor is a strict dimer at physiological receptor density in live neurons, but revealed the ability of this dimer to assemble into structurally distinct larger complexes upon stabilization of either the active or inactive state. This observation leads us to propose a role for the oscillation of these receptors between conformational states under basal conditions in limiting their oligomerization. In turn, linkage between ligation and oligomerization opens new perspectives on the role of allosteric communication between receptor oligomers in regulating their physiological function.

## Methods

### Materials

SNAP-Lumi4-Tb, SNAP-Green, SNAP-Red and BG-Alexa Fluor 488 (BG-Alexa488) were from Cisbio Bioassays (Codolet, France). BINA, LY341495, and LY379268, were purchased from Tocris Bioscience (Bristol, UK). Laminin, dichloro(1,10-phenanthroline)copper(II) (CuP) and salts for preparing buffers were from Sigma-Aldrich (St. Louis, MO, USA).

### Plasmids

The pRK5 plasmids encoding the wild type rat mGlu_2_, GABA_B1_ and GABA_B2_ receptors with HA-tag, Flag-tag and/or SNAP-tag inserted just after the signal peptide (HA-ST-mGlu_2_, HA-GABA_B1a/b_, HA-ST-GABA_B1a/b_, Flag-GABA_B2_, Flag-ST-GABA_B2_) under control of the CMV promoter were previously described^16,20,55^. Plasmids for controlling the number of SNAP-tags per mGlu_2_ dimer with and without the V699C mutation (mGlu_2_-C1_KKXX_, ST-mGlu_2_-C2_KKXX_, mGlu_2_-V699C-C1_KKXX_, ST-mGlu_2_-V699C-C2_KKXX_) were previously described^20,22^. Plasmid encoding truncated mGlu_2_ (HA-ST-mGlu_2_-Δ846) was obtained by introducing a STOP codon after Leu846 by QuikChange (Stratagene). Plasmids encoding mGlu_2_ mutated to be locked in the inactive conformation with and without the F756D mutation (HA-ST-mGlu_2_-515NGlyc-L698C-L729C and HA-ST-mGlu_2_-515NGlyc-L698C-L729C-F756D) were generated by subcloning and QuikChange from previously described mutations^22,26^. For neuron specific expression, the CMV promoter in pRK plasmids was exchanged with the synapsin-1 promoter (gift from B. Bettler). The plasmids encoding the high affinity glutamate transporter EAAC1 and the chimeric G protein Gαqi9 were previously described^56,57^.

### Neuron culture and transfection

Hippocampi from Sprague-Dawley rat embryos (Janvier Labs, Saint Berthevin, France) on embryonic day 18 (E18) were dissected, dissociated by treatment with liberase TL (Roche, Basel, Switzerland) and mechanical trituration and plated on Nunc Lab-Tek II chambered cover slides (Thermo Fisher Scientific, Boston, MA, USA) coated with polyornithine and laminin at a density of ∼300 neurons/mm^2^. Neurons were cultured in Neurobasal medium (Thermo Fisher Scientific) supplemented with 2% B-27 (Thermo Fisher Scientific), 100 U/ml Penicillin-Streptomycin (Thermo Fisher Scientific), 10 mM HEPES, and 0.5 mM GlutaMAX (Thermo Fisher Scientific). 0.5 mM L-glutamine was added when plating the cells. Half of the medium was exchanged weekly. After 10 days *in vitro* (DIV), neurons were transfected with Lipofectamine 2000 (Thermo Fischer Scientific). The medium was exchanged after 4 hours of incubation with the transfection reagent with 50% fresh medium and 50% medium conditioned by incubation with primary neurons. Transfected neurons were imaged at 14–17 DIV.

### SNAP-tag labeling, ligand stimulation and disulfide cross-linking for microscopy

SNAP-tagged receptors expressed in transfected neurons were labeled in imaging buffer (127 mM NaCl, 2.8 mM KCl, 1.1 mM MgCl_2_, 1.15 mM CaCl_2_, 10 mM D-glucose, 10 mM HEPES pH 7.3), supplemented with 0.3% (sN&B) or 1% (TR-FRET) BSA for 1 h at 37 °C followed by a wash in imaging buffer with 1% BSA and three washes in imaging buffer. For two-photon imaging and sN&B analysis receptors were labeled with 300 nM BG-Alexa488 and for TR-FRET microscopy receptors were labeled with 100 nM SNAP-Lumi4-Tb and 500 nM SNAP-Red. For experiments with ligands the neurons were incubated either with 10 nM LY379268 and 3 μM BINA, or with 400 nM LY341495, during labeling and imaging. Disulfide cross-linking was done by adding a final concentration of 150 μM CuP for the last 10 min of SNAP-tag labeling. For mGlu_2_ mutated to be locked in the inactive conformation the neurons were incubated with 400 nM LY341495 together with SNAP-tag labeling, i.e. 50 min before and during CuP incubation.

### Two-photon imaging and sN&B analysis

Images for sN&B analysis were acquired in imaging buffer on a two-photon scanning imaging setup with an Axiovert 200 M (Carl Zeiss Microscopy, Jena, Germany) inverted microscope stand with a Plan-Apochromat 63×, 1.4 NA oil-immersion objective (Carl Zeiss Microscopy) and an ALBA V laser scanning and detection system controlled using VistaVision 4.66 software (ISS, Champain, IL, USA). The excitation was performed at 940 nm with a Mai Tai femtosecond IR tunable laser (Spectra Physics, Santa Clara, CA, USA). Emitted light was detected with an AQR-14 avalanche photodiode (Perkin Elmer, Waltham, MA, USA) placed after a 530/43 nm bandpass filter. Axial drift was minimized using a homemade autofocus module based on monitoring and maintaining the position of the reflection of a near IR laser beam on the backside of the culture chamber. The position of the centroid of the reflection was calculated and maintained during the acquisition by changing the voltage input controlling the piezo holder of the objective (NanoF 100, Mad City Labs, Madison, WI, USA) using homemade software written in LabView (National Instruments, Austin, TX, USA).

For imaging of Alexa488 labeled receptors in transfected neurons, stacks of 100 images of 20 μm × 20 μm or 40 μm × 40 μm were acquired with a pixel size of 156 nm × 156 nm, a pixel bit depth of 16 bits, and a dwell time at each pixel of 40 μs. With a laser power at the sample level of 2 mW, the photobleaching was only 2 ± 1.4% for a 100 image stack, and therefore the brightness calculation was not biased. In addition, any image stack where photobleaching was above 10% was systematically removed from the analysis.

sN&B analysis was done in Igor Pro 6.36 (Wavemetrics, Lake Oswego, USA) using a custom script. The average intensity of the stack was calculated for each pixel and a threshold was applied to remove the background. In addition, out of focus pixels, mobile parts, and internalized vesicles were removed manually. The number and the molecular brightness values were extracted for each pixel of the image and used to construct the number map and the molecular brightness map, using

$$F=\langle F(t)\rangle $$

$$n=\frac{{F}^{2}}{\langle \delta F{(t)}^{2}\rangle -F}$$

$$\varepsilon =\frac{\langle \delta F{(t)}^{2}\rangle }{F}-1$$

where F(t) is the fluorescence intensity at time t, n is the number and *ε* is the molecular brightness. The histogram of the molecular brightness map was plotted and fitted using a Gaussian function. The peak value of the Gaussian represents the average molecular brightness of the receptors in the region of interest (ROI). Accumulated histograms were calculated based on all the molecular brightness values for a given expression range and fitted to multiple log-normal curves using the multi-peak fitting function in Igor Pro.

The molecular brightness of monomeric Alexa488 (*ε*_0_) was determined using a solution of 60 nM Alexa488 (Thermo Fisher Scientific) in 80% glycerol and 100 mM Tris buffer (pH 8). This value was used as the monomeric reference value for normalization of the molecular brightness of Alexa488 labeled receptors in transfected neurons, to determine the number of subunits per complex (*ε*/*ε*_0_) and to estimate the expression level of a ROI by dividing the median intensity by *ε*_0_.

### sdAb labeling, image acquisition and image analysis

Non-transfected or HA-ST-mGlu_2_ transfected hippocampal neurons were labeled with 100 nM DN1-d2^29,58^ (∼10× K_d_) in imaging buffer supplemented with 1% BSA for 1 h at 37 °C followed by a wash in imaging buffer with 1% BSA and three washes in imaging buffer.

Images of labeled neurons were acquired in imaging buffer on an Axio Observer Z1 (Carl Zeiss Microscopy) inverted microscope stand equipped with a Fluar 40×, 1.3 NA oil immersion objective (Carl Zeiss Microscopy) in epifluorescence mode. The d2 fluorophore was excited with a mercury lamp using a 620/60 nm bandpass filter and the emission was collected for 300 ms on an Orca R2 CCD camera (Hamamatsu Photonics, Hamamatsu, Japan) after a 700/75 nm bandpass filter. For quantification, labeled neurons were selected by thresholding background subtracted images using a custom script in ImageJ version 1.51f ^59^ followed by manual refinement of the selection to remove pixels not belonging to the cell. The mean d2 intensity was measured for this selection. To compare the d2 intensity per cell to the expression levels observed in sN&B the d2 intensity per cell was multiplied by the ratio of the mean expression per cell in sN&B experiments to the mean d2 intensity per cell for neurons transfected with HA-ST-mGlu_2_ and measured in the absence of ligands.

### TR-FRET microscopy and image analysis

Images were acquired in imaging buffer with a homebuilt TR-FRET microscope^37^ equipped with an α Plan-Apochromat 63×, 1.46 NA oil immersion objective (Carl Zeiss Microscopy). Briefly, the donor was excited with a 349 nm Nd:YLF pulsed laser at 300 Hz with ∼68 μJ/pulse followed by collection of either the donor signal using a 550/32 nm bandpass filter or the TR-FRET signal using a 700/75 nm bandpass filter. In both cases, images were acquired with 10 μs delay between excitation and collection of emission, 3 ms acquisition time and 4000 acquisitions. The acceptor was excited with a mercury lamp using a 620/60 nm bandpass filter and the emission was collected for 300 ms with a 700/75 nm bandpass filter. Donor and TR-FRET images were shading corrected by dividing the raw image with a background image generated for each image using the ‘Subtract background’ function in ImageJ. For quantification, labeled neurons were selected by thresholding the donor image using a custom script in ImageJ followed by manual refinement of the selection to remove pixels not belonging to the cell. The mean donor and TR-FRET intensities were measured for this selection. The background signals were subtracted and the mean TR-FRET intensity was corrected for donor bleedthrough (6%). Acceptor bleedthrough and direct acceptor excitation were not detected.

### Conformational TR-FRET sensor and inositol phosphate (IP) accumulation

HEK293 cells were cultured in Dulbecco’s modified Eagle’s medium (DMEM, Thermo Fischer Scientific) supplemented with 10% (vol/vol) fetal bovine serum (Sigma Aldrich) in a humid atmosphere at 37 °C and 5% CO_2_. Experiments were carried out in polyornithine-coated, black Cellstar 96-well plates (Greiner Bio-One, Kremsmünster, Austria). Absence of mycoplasma contamination was routinely confirmed with MycoAlert (Lonza, Basel, Switzerland). Cells were cotransfected with a plasmid encoding the glutamate transporter EAAC1 and medium was exchanged to DMEM with GlutaMAX (Thermo Fischer Scientific) at least two hours before the experiment to reduce ambient glutamate levels. For IP accumulation the cells were cotransfected with a plasmid encoding the chimeric G protein Gαqi9.

For the conformational TR-FRET sensor experiments, HEK293 cells were transfected by electroporation as previously described^56^. After 24 h cells were labeled with 100 nM SNAP-Lumi4-Tb and 60 nM SNAP-Green in Krebs buffer (10 mM Hepes pH 7.4, 146 mM NaCl, 4.2 mM KCl, 1 mM CaCl_2_, 0.5 mM MgCl_2_, 5.6 mM glucose, 0.1% BSA) for 1 h at 37 °C followed by four washes with Krebs buffer and the LRET was measured by TR-FRET as previously described^26^. During the labeling the cells were incubated with CuP as described for sN&B experiments.

For IP accumulation experiments, HEK293 cells were transfected with Lipofectamine 2000 (Thermo Fischer Scientific) using a reverse transfection protocol. 24–48 h after transfection cells were stimulated for 30 min and IP accumulation was measured with the IP-One HTRF kit (Cisbio) according to the manufacturer’s recommendations using a PHERAstar FS microplate reader (BMG Labtech, Ortenberg, Germany). For disulfide cross-linking cells were incubated with CuP as described for sN&B experiments prior to IP accumulation measurements.

### Statistical analysis

For sN&B analysis of receptor stoichiometry each data point corresponds to the peak value of a molecular brightness histogram of a ROI from a transfected neuron (Figs 1f, 2b,c, 4a–c, 6b–d and Supplementary Figs S1d, S2b,c, S5b–d, S8). Each data set is pooled from at least three independent experiments. In addition, scatter plots show the mean ± SD of the molecular brightness histogram peak values (Figs 2c, 4c, 6d and Supplementary Figs S5d, S8b,d). For sdAb labeling each data point represents the mean intensity from one neuron (Fig. 3c). In addition, the mean of these data points is shown. Experiments are pooled from four independent experiments. For TR-FRET microscopy each data point represents the mean intensity from one neuron (Fig. 5b). In addition, the mean ± SD of these data points is shown. Experiments are pooled from 2–4 independent experiments. For conformational TR-FRET sensor and IP accumulation experiments (Supplementary Figs S5a and S7) the results shown are the mean ± SEM of at least three independent experiments, each performed in triplicate. Comparison of values was done with an unpaired, two-sided t-test for two values or with one-way ANOVA followed by Bonferroni’s multiple comparisons test for more than two values using Prism 7 (GraphPad Software, San Diego, CA, USA). P < 0.05 was considered significant.

### Data avaibility

The datasets generated during the current study are available from the corresponding author on reasonable request.

## Electronic supplementary material

Supplementary Information
