# Supplementary material for: Oligomerization of a G protein-coupled receptor in neurons controlled by its structural dynamics
Source: Sci Rep. 2018 Jul 10;8:10414. doi: 10.1038/s41598-018-28682-6 (PMC6039492; doi:10.1038/s41598-018-28682-6)
Supplement: Supplementary file 1 — Supplementary Information [file 41598_2018_28682_MOESM1_ESM.pdf]

Supplementary Information

Oligomerization of a G protein-coupled receptor in neurons controlled by its structural dynamics

Authors:

Thor C. Møller[1 +], Jerome Hottin[2 +], Caroline Clerté[2], Jurriaan M. Zwier[3], Thierry Durroux[1], Philippe Rondard[1], Laurent Prézeau[1], Catherine A. Royer[2,4], Jean-Philippe Pin[1 *], Emmanuel Margeat[2 *], Julie Kniazeff[1]

1: IGF, Univ Montpellier, CNRS, INSERM, Montpellier, France.

2: CBS, Univ Montpellier, INSERM, CNRS, Montpellier, France.

3: Cisbio Bioassays, F-30200 Codolet, France.

4: Department of Biological Sciences, Rensselaer Polytechnic Institute, Troy NY 12180, USA.

+ these authors contributed equally to this work

*co-corresponding authors: jppin@igf.cnrs.fr; margeat@cbs.cnrs.fr

Supplementary Method

sN&B is one of a family of techniques based on fluorescence fluctuation analysis. In these approaches, fluorescent particles diffuse in and out of a small effective observation volume, V_{eff}, created inside the sample, yielding fluctuations in fluorescence intensity about the mean. Both the mean fluorescence, $\langle F \rangle$, and the variance, σ^2 (fluctuations) are calculated from the time trace of fluorescence intensity. As long as the dwell time is long enough to capture molecular fluorescence, but short enough to prevent averaging out of fluctuations due to diffusion, fluctuations follow Poisson statistics. The average number of fluorescent particles inside the excitation volume is inversely proportional to the amplitude of the fluctuations:

$$N = \langle F \rangle^2 / \sigma^2 \qquad (1)$$

while the ratio

$$B = \sigma^2 / \langle F \rangle \qquad (2)$$

is termed molecular brightness, and determines the average photon count per dwell-time per diffusing particle. Detector shot noise, that ensues from the quantum nature of light, contributes to artificially increase the signal variance such that *N* and *B* values must be shot-noise corrected to yield the true number of particles, *n*, in the effective excitation volume, V_{eff}[25],

$$n = (N \times B)/(B-1) \qquad (3)$$

and their true molecular brightness,

$$(\varepsilon = B-1) \qquad (4)$$

in counts per dwell-time per emitting particle.

In general, for two samples displaying identical average fluorescence intensity, a sample containing a few bright molecules will exhibit significantly larger fluctuations than a sample containing a large number of dim ones. Consequently, a GFP-fusion protein that dimerizes will yield

twice the molecular brightness (ε) of the corresponding monomers, as well as two-fold fewer diffusing particles (*n*) since they move as pairs. Then, the measured molecular brightness in the sample, normalized to that of the dye ε_{GFP}, is a direct measure of the protein stoichiometry. Simultaneously, the fluorescence intensity reports on the GFP-protein receptor expression level.

In scanning Number and Brightness (sN&B), multiple (e.g. 100) rapid raster scans of imaging fields of view (FOV) provide multiple fluorescence intensity measurements at each pixel of the FOV, from which the $<F>$ and σ^2 at each pixel can be calculated, yielding spatial maps of number, *n*, and brightness, ε. While single pixel-based sN&B data can be rather noisy, and include both positive and negative brightness values, averaging over all pixels within an individual region of interest (the cell membrane, for example) provides robust values for the average true brightness. Note that since measurements are made at each pixel only every 2-3 seconds (frame time), even very slowly moving particles (such as membrane proteins) diffuse and hence lead to fluctuations from one data point to the next. This key difference in acquisition timing compared to classical single point fluorescence correlation spectroscopy (FCS) makes sN&B much more useful than FCS in live cell measurements of membrane proteins.

Supplementary Fig. S1

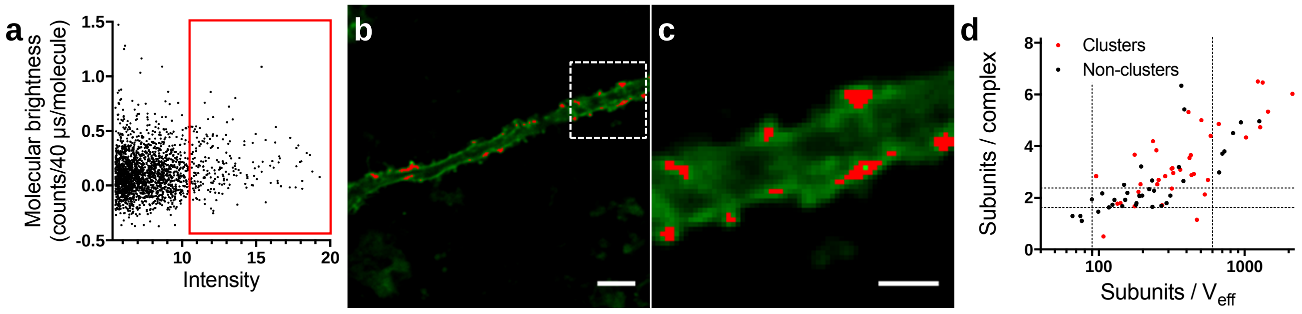

Supplementary Figure S1: **Spatial heterogeneity of $mGlu_2$ oligomerization. (a)** High intensity clusters are selected by taking the 10% pixels with the highest intensity (red square). **(b)** Intensity image with high intensity clusters highlighted in red. Scale bar represents 5 µm. **(c)** Magnification of dashed square in (b). Scale bar represents 2 µm. **(d)** Comparison of the subunits per complex for receptors in clusters (red) and receptors outside clusters (black).

Supplementary Fig. S2

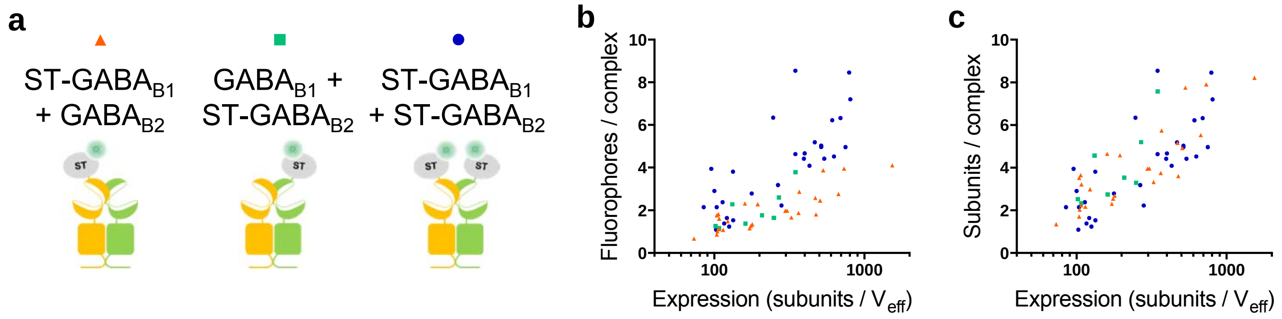

Supplementary Figure S2: The measured GABA$_B$ stoichiometry is independent of the labeling scheme. (a) Schematic representation of the three combinations of untagged and SNAP-tagged GABA$_B$ receptor subunits. **(b-c)** Number of fluorophores (b) or subunits (c) per complex as a function of the expression of subunits per effective volume (V_{eff}) for the different transfection combinations: ST-GABA$_{B1}$ + GABA$_{B2}$, (orange), GABA$_{B1}$ + ST-GABA$_{B2}$ (green), ST-GABA$_{B1}$ + ST-GABA$_{B2}$ (blue).

Supplementary Fig. S3

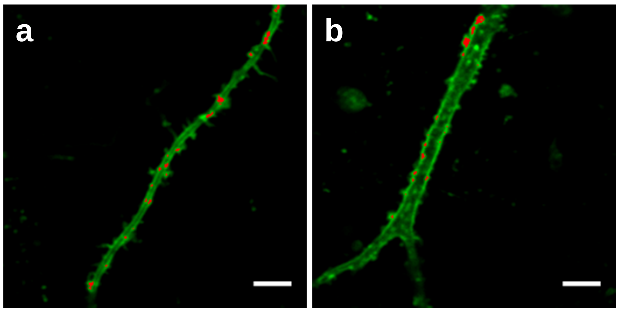

Supplementary Figure S3: **Influence of ligands on spatial heterogeneity.** (**a-b**) Two-photon intensity images with high intensity clusters highlighted in red from neurons transfected with ST-mGlu$_2$ and bound to either agonist and PAM (a) or antagonist (b). Scale bars represent 5 µm.

Supplementary Fig. S4

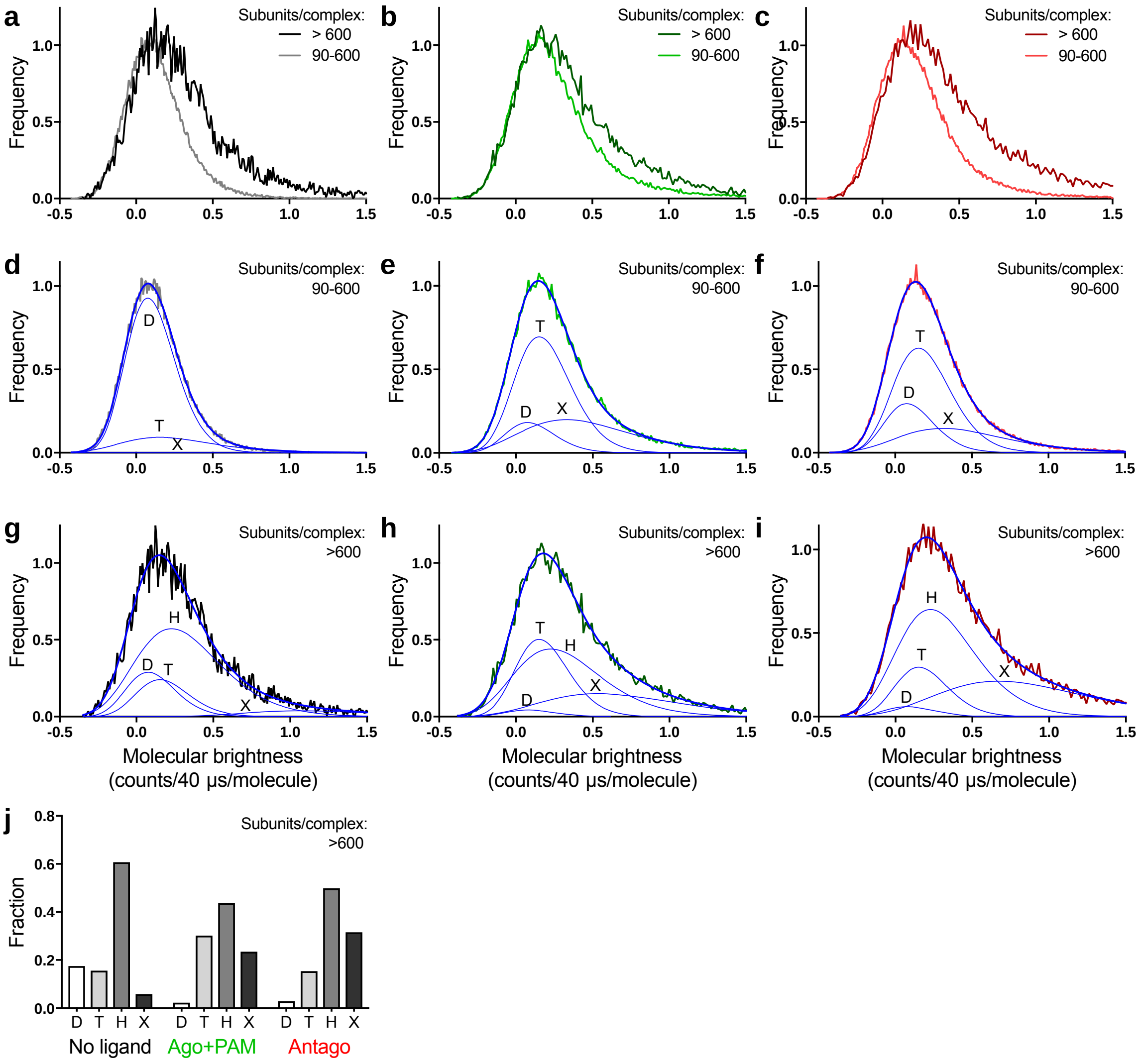

Supplementary Figure S4: **Heterogeneity of $mGlu_2$ oligomerization state. (a-c)** Comparison between the accumulated molecular brightness histograms for ST-$mGlu_2$ transfected neurons with expression in the 90-600 subunits/V_{eff} range (grey, light green, light red) or above 600 subunits/V_{eff} (black, dark green, dark red) for receptor in the absence of ligand (a), bound to agonist and PAM (b) or bound to antagonist (c). **(d-i)** Log-normal fits of the accumulated histograms for $mGlu_2$ in absence of ligand at expression in the 90-600 subunits/V_{eff} range (d) and above 600 subunits/V_{eff} (g), for $mGlu_2$ bound to agonist and PAM at expression in the 90-600 subunits/V_{eff} range (e) and above 600 subunits/V_{eff} (h), and for $mGlu_2$ bound to antagonist at expression in the 90-600 subunits/V_{eff} range (f) and above 600 subunits/V_{eff} (i). The components of the total fit (thick blue curve) corresponding to the populations of dimers (D), tetramers (T), hexamers (H) and complexes larger than tetramers (X) are shown with thin blue curves. The hexamer population was only included for expression above 600 subunits/V_{eff}. **(j)** Fraction of the different oligomeric species for neurons with expression above 600 subunits/V_{eff}.

Supplementary Fig. S5

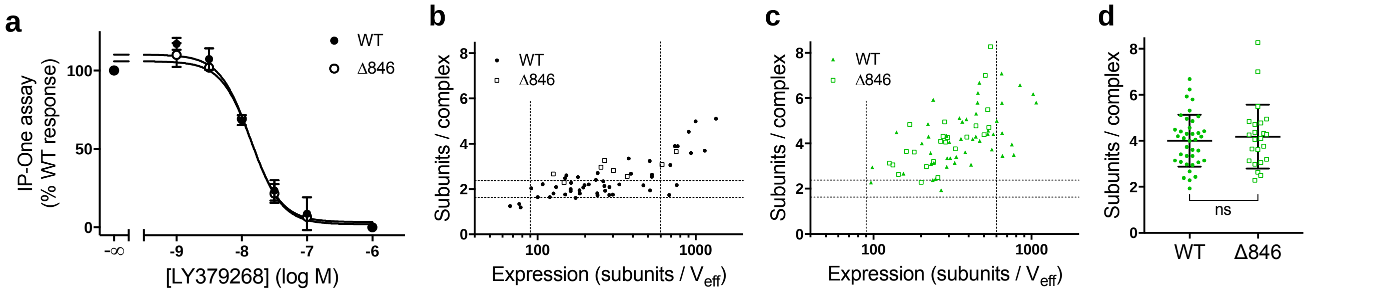

Supplementary Figure S5: The C-terminal domain of mGlu$_2$ receptor does not influence its oligomerization. The amino acids after position 846 in the C-terminal domain of the mGlu$_2$ receptor are deleted (mGlu$_2$-Δ846) to test their role in mGlu$_2$ receptor oligomerization. (**a**) The functionality of the mutated receptor is identical to wild type ST-mGlu$_2$, as tested by measuring accumulation of the second messenger inositol monophosphate (IP1) in HEK293 cells. Results are mean ± SEM of three independent experiments performed in triplicates. (**b-c**) Comparison between the number of subunits per complex in ST-mGlu$_2$ (filled circles) and ST-mGlu$_2$-Δ846 transfected neurons (open circles) in absence of ligand (b) or bound to agonist and PAM (c) as a function of the expression level. (**d**) Comparison between the number of subunits per complex in ST-mGlu$_2$ and ST-mGlu$_2$-Δ846 transfected neurons bound to agonist and PAM for neurons in the 90-600 subunits/V$_{eff}$ expression range. The mean stoichiometry of ST-mGlu$_2$-Δ846 (4.2 ± 1.4 subunits/complex, mean ± SD) is not statistically different from wild type ST-mGlu$_2$ (4.0 ± 1.1 subunits/complex) (unpaired, two-sided t-test). Error bars represent SD.

Supplementary Fig. S6

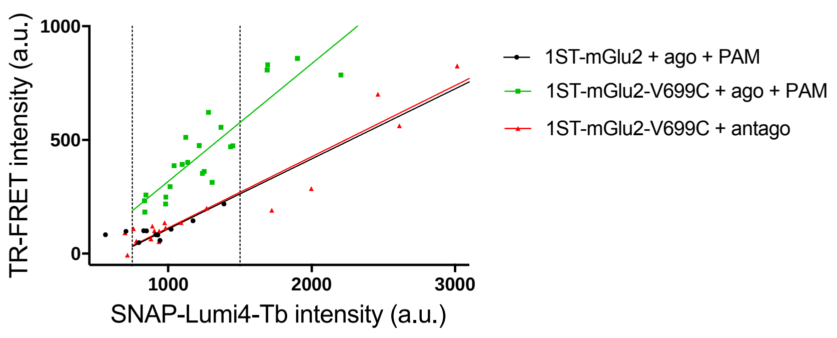

Supplementary Figure S6: Dimer-dimer TR-FRET as a function of the receptor expression level. TR-FRET between $mGlu_2$ receptors with one SNAP-tag per dimer and the V699C mutation that allows crosslinking of $mGlu_2$ dimers when the receptor is stabilized in the active state as a function of the expression level estimated by the donor (SNAP-Lumi4-Tb) intensity.

Supplementary Fig. S7

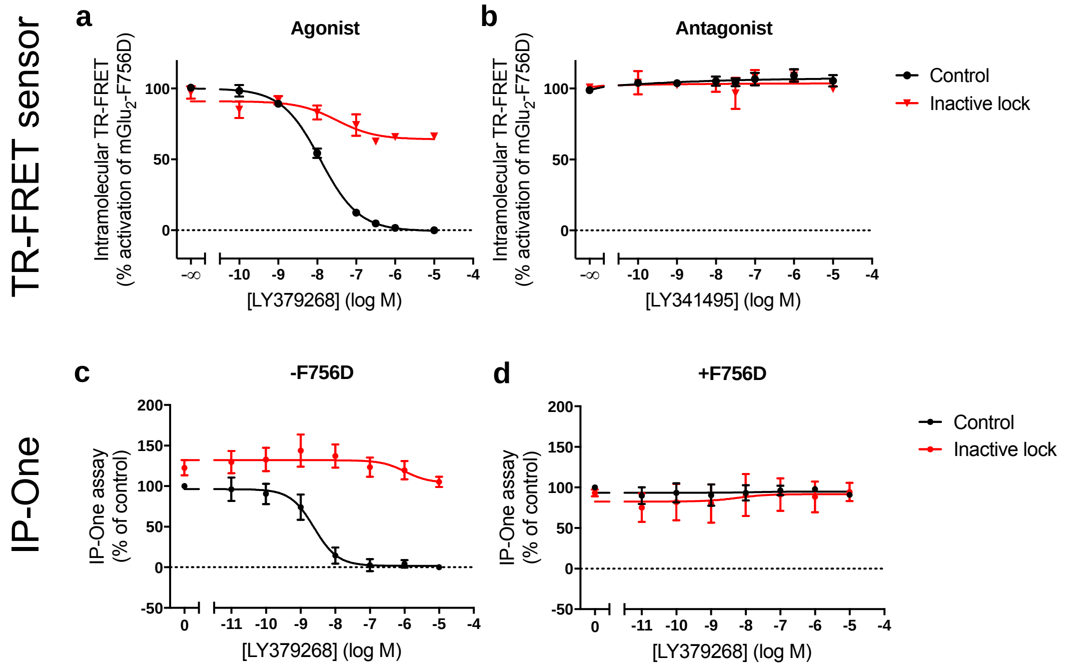

Supplementary Figure S7: $mGlu_2$ 'inactive lock' mutant is conformationally constrained to the inactive state. (**a-b**) Conformational TR-FRET sensor experiments with $mGlu_2$ mutant locked in the inactive conformation (inactive lock) compared to wild type $mGlu_2$ (control) in HEK293 cells. The conformational changes in the extracellular domains of the $mGlu_2$ dimer are monitored via the change in TR-FRET between N-terminal SNAP-tags labeled with SNAP-Lumi4-Tb and SNAP-Green. When $mGlu_2$ is activated the N-termini of the dimer move away from each other, which leads to decreased TR-FRET in the conformational sensor. (**a**) Dose-response curve of LY379268, a full agonist for $mGlu_2$. (**b**) Dose-response curve of LY341495, a competitive antagonist for $mGlu_2$. (**c-d**) Functional experiments monitoring IP1 accumulation for agonist activated $mGlu_2$ locked in the inactive conformation without (c) and with (d) the F756D mutation that prevents the receptor from activating G protein compared to wild type $mGlu_2$ (control). Results are mean ± SEM of three independent experiments performed in triplicates.

Supplementary Fig. S8

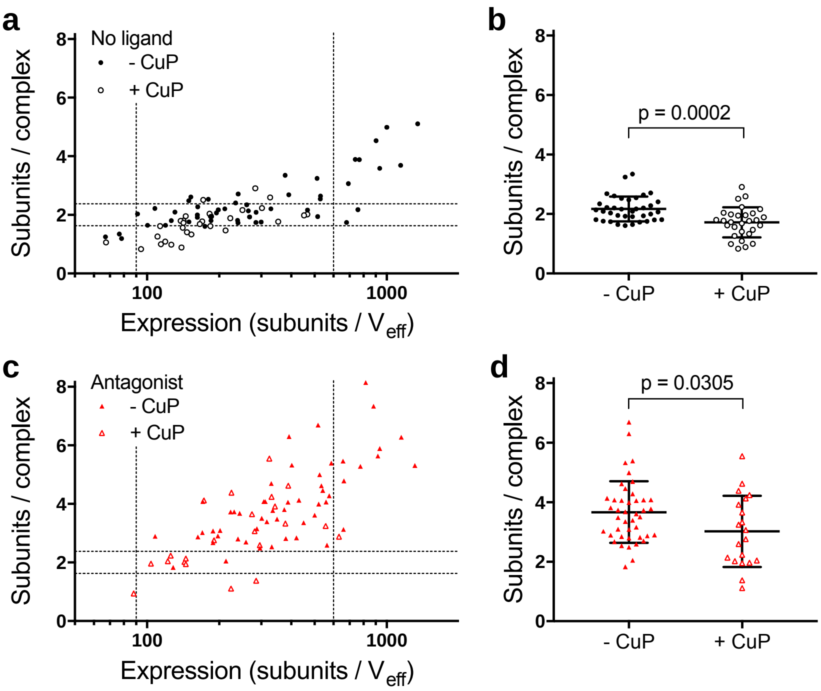

Supplementary Figure S8: Copper phenanthroline (CuP) reduces apparent mGlu$_2$ receptor stoichiometry. mGlu$_2$ receptor subunits per complex in ST-mGlu$_2$ transfected neurons either untreated (filled circles) or treated with CuP (open circles) as a function of subunits per effective volume (V_{eff}) (**a,c**) or for cells with an expression between 90 and 600 subunits/V_{eff} (**b,d**). The measurements were performed either in absence (**a-b**) or presence of a saturating concentration of competitive antagonist (400 nM LY341495). Error bars represent SD.
